# Terpenes From the Root of *Salvia hypoleuca* Benth

**DOI:** 10.1186/2008-2231-20-66

**Published:** 2012-10-24

**Authors:** Soodabeh Saeidnia, Mitra Ghamarinia, Ahmad R Gohari, Alireza Shakeri

**Affiliations:** 1 Medicinal Plants Research Center, Tehran University of Medical Sciences, P. O. Box 14155–6451, Tehran, Iran; 2 Department of Chemistry, Faculty of Science, Golestan University, Gorgan, Iran; 3 Medicinal Plants Research Center, Faculty of Pharmacy, Tehran University of Medical Sciences, PO Box 14155–6451, Tehran, Iran

**Keywords:** *Salvia hypoleuca*, Coleonolic acid, 7α-acetoxyroyleanone, 3-epimaslinic acid, 3-epicorosolic acid, Manool

## Abstract

**Background:**

The genus *Salvia*, with nearly 900 species, is one of the largest members of Lamiaceae family. In the Flora of Iran, the genus *Salvia* is represented by 58 species of which 17 species are endemic. *Salvia hypoleuca* Benth., is one of these species growing wildly in northern and central parts of Iran. *Salvia* species are well known in folk medicine and widely used for therapeutic purposes. Literature review shows that there is no report on phytochemical investigation of the roots of *S. hypoleuca*.

**Results:**

The separation and purification process were carried out using various chromatographic methods. Structural elucidation was on the basis of NMR and MS data, in comparison with those reported in the literature. The isolated compounds were identified as sitosteryl oleate (1), β-sitosterol (2), stigmasterol (3), manool (4), 7α-acetoxy royleanone (5), ursolic acid (6), oleanolic acid (7), 3-epicorosolic acid (8), 3-epimaslinic acid (9) and coleonolic acid (10).

**Conclusions:**

In the present study, three sterols, two diterpenes and five triterpenes were isolated from the ethyl acetate extract of the roots of *S. hypoleuca*. As the chemotaxonomic significance, some of the isolated compounds (1–7, 9) have not been previously reported from the species *S. hypoleuca*, while the triterpenes 8 and 10 are now documented from *Salvia* genus for the first time.

## Background

The genus *Salvia* L. (Lamiaceae), with more than 900 species throughout the world, is represented 58 species in Iran, 17 of which are endemic. Most of the species are used as herbal tea and flavoring agent by people and also used in traditional medicine as tonic, antirheumatoid, antimicrobial and carminative [1-3]. *Flora Orientalis* includes as many as 107 species of *Salvia*[4]. *Salvia hypoleuca* Benth., is one of these species which growing wildly in northern and central parts of Iran [1].

Literature review show that various secondary metabolites such as terpenoids, phenolic acids [5], polyphenols, flavonoids [3,6] and anthocyanins [7] have been reported from *Salvia* species. Limonene, α-pinene, β-pinene, 1,8-cineol, bicyclogermacrene, caryophyllene oxide and α-gurjunene are the main components of the essential oils of various species of *Salvia* growing wildly in Iran [8-11]. In the literature, there are several reports on phytochemical investigation of the above mentioned species.

Several sesterterpene lactones, isomeric epoxides, monolactone and hypoleuenoic acid have been reported from varies fractions of *S. hypoleuca*[12-14]*.* The main aromatic components of the essential oil of *S. hypoleuca* roots have been identified as hexadecanoic acid (27.4%) and viridiflorol (14.9%) [15], while germacrene D (15.1%) and β-caryophyllene (22.0%) identified as the major constituents during flowering stages [16]. A great number of diterpenes exhibited interesting biological activities *e.g.* anti-tuberculous, antitumour, antibacterial, antileishmanial and antispasmolytic, and *Salvia* species are the excellent source of diterpenoids [17]. In this study, we aim to report the isolation and identification of some sterols, diterpenoids and triterpenoids from the root extract of *S. hypoleuca* which have not been previously reported from this species.

## Methods

### Instruments and materials

^1^H-NMR and ^13^C-NMR spectra were recorded on a Brucker Avance 500 DRX spectrometer ® with tetramethylsilane as an internal standard and chemical shifts are given in δ (ppm). Multiple-pulse experiments (HSQC, HMBC and H-H COSY) were performed using the standard Bruker ® programs. Silicagel 60 F_254_ and Silicagel 60 RP-18 F_254_S pre-coated plates (Merck ®) were used for TLC. The spots were detected by spraying with anisaldehyde-H_2_SO_4_ reagent followed by heating.

### Plant materials

The roots of *Salvia hypoleuca* Benth., were collected from Tehran province (near to Damavand city), Iran, at flowering stage in August 2008 and dried at room temperature. Voucher specimen was deposited at the Herbarium of Complex of Academic Center for Educational and Cultural Research under number ACECR-266.

### Extraction and isolation process

Dried roots of *S. hypoleuca* (900 g) were cut into small pieces and extracted with ethyl acetate at room temperature by percolation method for 72 hours and 3 times. The solvent was evaporated by rotary evaporator. The ethyl acetate extract (2 g) was fractionated by silica gel column chromatography (CC) with hexane, hexane: chloroform (9:1, 5:5), ethyl acetate and methanol, to give seven fractions (A-G). Fraction A (88 mg) was subjected to silica gel CC with hexane: ethyl acetate (19:1) to obtain compound 1 (21 mg). Fraction B (200 mg) was submitted to silica gel CC with hexane: ethyl acetate (9:1) to give compound 2 and 3 (17 and 13 mg respectively). Fraction C (134 mg) was submitted to silica gel CC with hexane: ethyl acetate (19:1) to result in six fractions (C_1_-C_6_). Fraction C_5_ (14 mg) was chromatographed on silica gel CC with chloroform: ethyl acetate (19:1) to yield compound 4 (8 mg). Fraction D (126 mg) was fractionated on silica gel CC with hexane: ethyl acetate (19:1) to obtain six parts (D_1_-D_6_). Fraction D_3_ (27 mg) was separated on sephadex LH_20_ with methanol: ethyl acetate (7:3) to gain four fractions (D_31_-D_34_). Fraction D_33_ (10 mg) was subjected to reverse phase (RP) silica gel CC with methanol: water (8:2) to result in compound 5 (5 mg). Fraction F (624 mg) was fractionated on silica gel CC with chloroform: methanol (19:1) to yield three parts (F_1_-F_3_). Fraction F_1_ (204 mg) was chromatographed on silica gel CC with chloroform: ethyl acetate (8:2) to obtain nine fractions (F_11_-F_19_). Fraction F_13_ (30 mg) was subjected to sephadex LH_20_ with methanol to result in compound 6 and 7 (7 and 5 mg, respectively). Fraction F_17_ (8 mg) was submitted to sephadex LH_20_ with methanol to obtain compound 8 and 9 (3 and 2 mg, respectively). Fraction F_2_ (67 mg) was further isolated on RP silica gel CC with methanol: water (9:1) to give compound 10 (2 mg).

## Results

**Figure 1:**
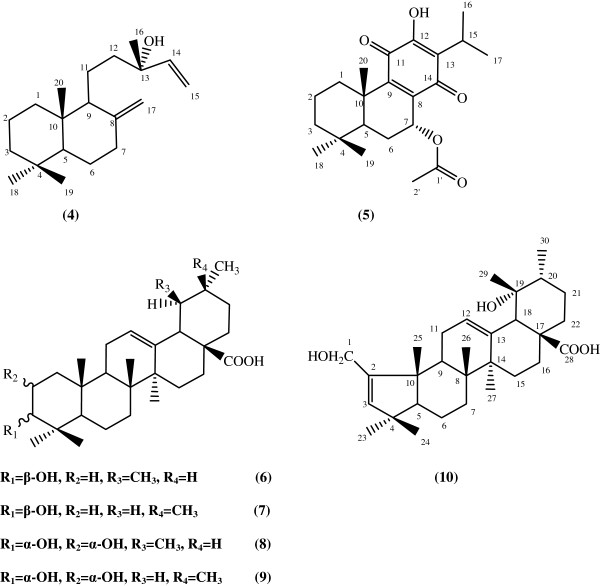
**Structures of the isolated terpenes from the root of *Salvia hypoleuca.***

**Figure 2:**
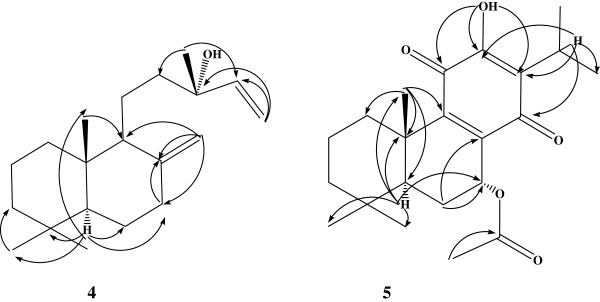
HMBC correlations and important assignments of the compounds 4 and 5 (H→C).

In the present study, the ethyl acetate extract of the root of *S. hypoleuca* was used for the isolation process and structural elucidation was carried out based on spectral data. Three sterols, sitosteryl oleate (1) [18], β-sitosterol (2) [19] and stigmasterol (3) [20], two diterpenes, manool (4) [21] and 7α-acetoxyroyleanone (5) [22] together with five triterpenes, ursolic acid (6) [19], oleanolic acid (7) [23], 3-epicorosolic acid (8) [24], 3-epimaslinic acid (9) [25] and coleonolic acid (10) [26], (Figure 1) were isolated and identified by comparison of their spectral data (^1^H-NMR, ^13^C-NMR, HMBC, HSQC, ^1^H-^1^H COSY, EI-MS) with those reported in the literature. Because these compounds were previously published from other plant sources, we do not explain the spectral assignments here. NMR data (^1^H-NMR, ^13^C-NMR, HMBC, HSQC and DEPT) of the compound 4 and 5 in CDCl_3_ are shown in Tables 1 and 2 respectively. ^13^C-NMR data of the compounds 6–10 are indicated in Table 3. Also, HMBC correlations and important assignments of the compounds 4 and 5 (H→C) are appeared in Figure 2.

**Table 1:** **NMR data of the compound 4 in CDCl**_**3**_

**Carbon Number**	**DEPT**	**HSQC**		**HMBC**	**H-HCOSY**
		^**1**^**H-NMR δ(ppm)**	^**13**^**C-NMR δ(ppm)**		
**1**	CH_2_	1.00 (*m*, 1H)	39.07	C-5	H-1b, H-1a
		1.76 (*m*, 1H)			
**2**	CH_2_	1.36 (*m*, 1H)	17.70		H-2b, H-2a
		1.55 (*m*, 1H)			
**3**	CH_2_	1.17 (*m*, 1H)	42.19	C-4, C-5, C-18	H-3b, H-3a
		1.36 (*m*, 1H)		C-2	
**4**	C	-	33.16		-
**5**	CH	1.08 (*brd*, *J*=12.3 Hz, 1H)	55.58	C-4, C-6, C-7, C-18, C-20	-
**6**	CH_2_	1.76 (*m*, 2H)	24.42		-
**7**	CH_2_	1.95 (*m*, 1H)	38.35	C-6, C-8, C-17	-
		2.37 (*brd*, *J*=12.4 Hz, 1H)		C-5, C-6, C-8, C-9, C-17	
**8**	C	-	148.69		-
**9**	CH	1.55 (*m*, 1H)	57.32	C-8, C-10, C-17	-
**10**	C	-	39.87		-
**11**	CH_2_	1.48 (*m*, 1H)	19.38	-	H-11b, H-11a
		1.55 (*m*, 1H)		C-9, C-10, C-12	
**12**	CH_2_	1.27 (*m*, 1H)	41.43	C-16	H-12b, H-12a
		1.76 (*m*, 1H)		C-13, C-14	
**13**	C	-	73.58		-
**14**	CH	5.92 (*dd*, *J*=17.3,10.7 Hz, 1H)	145.29	C-13	H-15
**15**	CH_2_	5.04 (*d*, *J*=10.6 Hz, 1H)	111.52	C-13	H-14
		5.20 (*d*, *J*=17.3 Hz, 1H)		C-13, C-14	
**16**	CH_3_	1.27 (*s*, 3H)	27.66	C-12, C-14	-
**17**	CH_2_	4.51 (*s*, 1H)	106.45	C-7, C-8, C-9	-
		4.81 (*s*, 1H)		C-7, C-9	
**18**	CH_3_	0.79 (*s*, 3H)	21.71	C-3, C-4, C-5	-
**19**	CH_3_	0.86 (*s*, 3H)	33.62	C-3, C-4, C-5, C-18	-
**20**	CH_3_	0.67 (*s*, 3H)	14.43	C-9	-

**Table 2:** **NMR data of the compound 5 in CDCl**_**3**_

**Carbon number**	**DEPT**	**HSQC**		**HMBC**
		^**1**^**H-NMR δ(ppm)**	^**13**^**C-NMR δ(ppm)**	
**1**	CH_2_	1.20 (*m*, 1H)	35.77	
		2.72 (*brd*, *J*=13.0 Hz, 1H)		
**2**	CH_2_	1.58 (*m*, 1H)	18.80	C-10
		1.72 (*dd*, *J*=13.3,13.4 Hz, 1H)		
**3**	CH_2_	1.21 (*m*, 1H)	40.97	-
		1.47 (*brd*, *J*=12.7 Hz, 1H)		C-18, C-19
**4**	C	-	32.96	
**5**	CH	1.47 (*brd*, *J*=12.7 Hz, 1H)	46.12	C-4, C-7, C-10, C-18, C-20
**6**	CH_2_	1.60 (*m*, 1H)	24.61	C-5, C-10
		1.93 (*d*, *J*=14.9 Hz, 1H)		C-5, C-7, C-8, C-10
**7**	CH	5.92 (*brs*, 1H)	64.48	
**8**	C	-	139.45	
**9**	C	-	149.94	
**10**	C	-	39.06	
**11**	C	-	183.72	
**12**	C	-	150.75	
**13**	C	-	124.66	
**14**	C	-	185.45	
**15**	CH_3_	3.15 (*m*, 1H)	24.15	C-12, C-13, C-14, C-17
**16**	CH_3_	1.17 (*d*, *J*=7.0 Hz, 3H)	19.68	C-13, C-15, C-17
**17**	CH_3_	1.22 (*d*, *J*=7.0 Hz, 3H)	19.86	C-13, C-15, C-16
**18**	CH	0.87 (*s*, 3H)	21.61	C-3, C-5, C-19
**19**	CH_3_	0.87 (*s*, 3H)	32.96	C-3, C-5, C-18
**20**	CH_3_	1.23 (*s*, 3H)	18.48	C-1, C-5, C-9, C-10
**1´**	C	-	169.46	
**2´**	CH_3_	2.03 (*s*, 3H)	21.11	C-1**´**
**OH-12**		7.12 (*s*, 1H)	-	C-11, C-12, C-13

**Table 3:** ^**13**^**C-NMR data of the compounds 6–10**

**Carbon Number**	**8**^**a**^	**9**^**a**^	**10**^**b**^	**Carbon Number**	**8**	**9**	**10**
**1**	41.94	41.69	61.4	**16**	24.13	23.27	27.3
**2**	66.49	66.50	156.1	**17**	48.10	46.48	-
**3**	78.91	78.92	135.2	**18**	52.63	41.04	55.3
**4**	38.34	38.34	42.7	**19**	39.04	45.89	73.5
**5**	48.13	48.14	64.4	**20**	38.84	30.67	44.4
**6**	18.03	18.03	18.3	**21**	30.61	33.83	26.6
**7**	32.73	32.46	35.3	**22**	36.70	32.46	39.0
**8**	39.49	39.70	42.9	**23**	28.48	28.48	30.3
**9**	47.28	47.35	43.1	**24**	21.81	21.79	21.9
**10**	38.23	38.42	51.8	**25**	16.47	16.33	19.1
**11**	23.27	22.95	27.1	**26**	16.98	17.13	16.5
**12**	125.64	122.46	129.5	**27**	23.74	26.07	25.5
**13**	138.06	143.68	140.3	**28**	181.95	181.95	182.2
**14**	42.10	41.76	43.3	**29**	17.10	33.05	27.7
**15**	27.96	27.63	29.9	**30**	21.16	23.58	16.5

^**a**^ In CDCl_3_.

^**b**^ In CD_3_OD.

The mass data of the compounds 1, 2, 3, 6 and 7 have been previously reported [27,28]. The mass of other compounds are followed: Manool (4): EIMS (70eV) *m/z*: 290 [M]^+^ (8), 272 (40), 204 (20), 257 (58), 189 (28), 137 (100), 121 (48), 95 (67). 3-epicorosolic acid (8): 472 [M]^+^ (5), 248 (100), 223 (18), 203 (61), 189 (13), 133 (20), 119 (10). 3-epimaslinic acid (9): 472 [M]^+^ (4), 248 (100), 235 (9), 223 (12), 203 (54), 189 (15), 133 (28). coleonolic acid (10): *m/z* 470 [M] ^+^ (7), 452 (25), 264 (18), 206 (15), 201 (35), 159 (28), 146 (50), 105 (100).

β-sitosterol: ^13^C-NMR (125 MHz, CDCl_3_): *δ*_*C*_ (from C-1 to C-29) 37.3, 31.7, 71.8, 42.3, 140.8, 121.7, 31.9, 31.9, 50.2, 36.5, 21.1, 39.8, 42.3, 56.8, 24.3, 28.3, 56.1, 11.9, 19.8, 36.2, 18.8, 34.0, 26.1, 45.8, 29.2, 19.0, 19.4, 23.1, 12.0.

Stigmasterol: ^13^C-NMR (125 MHz, CDCl_3_): *δ*_*C*_ (from C-1 to C-29) 37.3, 31.7, 71.8, 42.2, 140.8, 121.7, 31.9, 31.9, 50.2, 36.4, 21.1, 39.7, 42.2, 56.9, 24.4, 28.9, 56.0, 12.0, 19.4, 40.5, 21.2, 138.3, 129.3, 51.6, 31.9, 19.0, 21.1, 25.4, 12.2.

## Discussion

Literature reviews show that *Salvia* species are important medicinal and food plants. About 200 triterpenoids have been isolated and identified from about 100 *Salvia* species and presented in a review article by Topcu [29]. The oleanane, and ursane triterpenes display various pharmacological activities. These triterpenes can be considered as the lead compounds for the development of new multi-targeting bioactive agents [30]. Both oleanolic and ursolic acid have been documented to protect liver against chemically induced injuries in laboratory animals *via* inhibition of toxicant activation and enhancement of immune systems. These two triterpenes have also been long-recognized as anti-inflammatory and anti-hyperlipidemic agents. Furthermore, anti-tumor activity has been noted from both non-toxic compounds [31].

Corosolic acid, a triterpenoid compound has been proved to have anti-diabetic effects on animal and human *via* enhancing glucose uptake in L6 myotubes and facilitating glucose transporters isoform 4 translocation in CHO/hIR cells. In addition, corosolic acid has been reported to inhibit the enzymatic activity of several non-receptor protein tyrosine phosphatases (PTPs) [32]. The abietane diterpene 7 α-acetoxy-royleanone, containing quinone moiety in its structure, was demonstrated to possess cytotoxic activity on cancer cell lines and also alkylating properties using the nucleophile 4-(4-nitrobenzyl) pyridine [33]. Among the reported antimicrobial labdane-type diterpenes, manool is the most active, since it furnished very promising MIC values for several tested bacteria that are closely associated with periodontitis [34].

According to chemotaxonomic significance, the isolated terpenes (manool (4), 7α-acetoxy-royleanone (5), ursolic acid (6), oleanolic acid (7), 3-epimaslinic acid (9)) were previously reported from other *Salvia* species such as *S. sclarea*[21], *S. pubescens*[35], *S. lavandulifolia*[36] and *S. officinalis*[37]. To the best of our knowledge, there is no report about the presence of the above mentioned compounds from *S. hypoleuca.* The triterpene 3-epicorosolic acid (8) and coleonolic acid (10) has not been reported from *Salvia* species, while some other genus of Lamiaceae such as *Perilla frutescens*[38] and *Coleus forskohlii*[39] contains these triterpenes.

## Conclusions

In conclusion, the results of this study indicated the presence of ten terpenes and sterols in the root extract of *S. hypoleuca* as: sitosteryl oleate (1), β-sitosterol (2), stigmasterol (3), manool (4), 7α-acetoxy royleanone (5), ursolic acid (6), oleanolic acid (7), 3-epicorosolic acid (8), 3-epimaslinic acid (9) and coleonolic acid (10). Some of the isolated compounds (1–7, 9) have not been previously reported from *S. hypoleuca* and the triterpenes 8 and 10 not reported from *Salvia* genus until now. The above mentioned compounds have been recognized as the biologically and pharmacologically active constituents from this medicinal and aromatic species of *salvia*.

## Competing interests

The authors declare that they have no competing interests.

## Authors' contributions

SS carried out the interpretation of the NMR data and identification of the compounds. MG carried out the isolation and purification process. ARG participated in design of the study, helped in structured elucidation and final approved of the version to be published. AS participated in drafting the manuscript and helped in isolation of the compounds. All authors read and approved the final manuscript.
